# Wie finanzieren wir die Corona-Schulden? Versuch einer „richtigen“ Antwort auf eine „falsche“ Frage aus Sicht der Modern Monetary Theory

**DOI:** 10.1007/s10273-021-2874-9

**Published:** 2021-03-17

**Authors:** Dirk Ehnst, Michael Paetz

**Affiliations:** 1 Berlin, Deutschland; 2 Fakultät für Wirtschafts- und Sozialwissen., Universität Hamburg, Von-Melle-Park 5, 20146 Hamburg, Deutschland

**Keywords:** B52, E12, E6

## Abstract

Das Jahr 2020 war geprägt von der COVID-19-Pandemie und ihren wirtschaftlichen Folgen. In Deutschland stiegen staatliches Defizit sowie die Schuldenquote infolge des Rückgangs der Wirtschaftsleistung auf geschätzt 5 % bzw. 75 % des BIP an. Um die wirtschaftliche Erholung von der Pandemie nicht durch die Rückkehr zu einem rigiden Sparkurs zu gefährden, ist es jetzt von besonderer Bedeutung, sich von falschen Vorstellungen bezüglich der Finanzierung sowie der Nachhaltigkeit staatlicher Ausgabenüberschüsse zu verabschieden. Nur so können die Weichen für eine Wirtschaftspolitik des 21. Jahrhunderts richtig gestellt werden.

## Abstract (en)

The year 2020 was marked by the COVID-19 pandemic and its economic consequences. In Germany, government deficits as well as the debt ratio rose to an estimated 5 % and 75 % of GDP respectively as a result of the decline in economic output. In order not to jeopardise the post-pandemic economic recovery by returning to a rigid austerity policy, it is now of particular importance to abandon misconceptions regarding the financing as well as the sustainability of government expenditure surpluses. This is the only way to set the right course for an economic policy of the 21st century.

Die Frage der Finanzierung der Staatsverschuldung zielt darauf ab, ob der Anstieg der Schulden langfristig zu einem Problem werden könnte, sofern man die Schulden in Zukunft nicht zurückzahlen bzw. durch neue Schulden ersetzen kann. Dieses Denken basiert im Kern auf der Theorie, nach der sich ein Staat finanzieren kann, indem er 1. Steuern einnimmt, 2. Staatsanleihen emittiert oder 3. seine Ausgaben von der Zentralbank bezahlen lässt. Allerdings sichert lediglich Fall 1 in der klassischen Theorie eine (langfristig) nachhaltige Staatsfinanzierung. Fall 2 kann bei zu hohen Ausgaben zu einem explosiven Pfad der Staatsschuldenquote führen. Bei steigender Schuldenquote würden private Investor:innen immer höhere Zinsen verlangen, die durch eine weitere Schuldenaufnahme gedeckt werden müssten. Wird eine Zahlungsunfähigkeit befürchtet, wird der Staat kein Geld mehr bekommen und eine Insolvenz wäre unausweichlich. Die Finanzierung über die Zentralbank (Fall 3) würde angeblich zu einer sicheren Hyperinflation führen.

**Dr. Dirk Ehnts** lehrte an der Europa-Universität Flensburg und an der TU Chemnitz.

**Dr. Michael Paetz** ist wissenschaftlicher Mitarbeiter für Lehraufgaben am Fachbereich Volkswirtschaftslehre der Universität Hamburg.

Heute wissen wir, dass die Verdreifachung des staatlichen Defizits der USA von etwa 1 Billion US-$ (2019) auf 3 Billionen US-$ (2020) jedoch völlig reibungslos verlief. Das staatliche Defizit der USA im zweiten Quartal 2020 betrug dabei 27,5 %. Die Verzinsung von Staatsanleihen fiel, die Inflation blieb niedrig und der Außenwert des US-Dollars nahm zu. Offenbar hat der Anstieg der Staatsverschuldung zu keinem der befürchteten Probleme geführt.

Diese Entwicklung steht im Einklang mit den Ideen der Modern Monetary Theory (MMT).[1] Sie sieht das staatliche Defizit als eine rein statistische Größe an, die das Ergebnis der wirtschaftlichen Aktivitäten ist und daher nicht zur Zielgröße gemacht werden sollte. Dies gilt auch, weil der Staat die ihm zufließenden Steuereinnahmen ohnehin nicht direkt kontrollieren kann. In der Corona-Pandemie fielen diese deutlich geringer aus als erwartet wurde.

### Modern Monetary Theory

Die MMT wurde vor ziemlich genau 25 Jahren vom US-amerikanischen Investor und Rennwagenkonstrukteur Warren Mosler aus der Taufe gehoben. Seine wesentliche Einsicht war, dass eine moderne Währung ein staatliches Monopol ist. Dieses ist heute meist an (staatliche) Zentralbanken übertragen, die als Bank des Staates fungieren. Sie bezahlen die Rechnungen der Regierung, indem sie das Guthaben von Banken bei der Zentralbank erhöhen

1 Elemente der MMT finden sich unter anderem in post-keynesianischen Publikationen wie Helmedag (2018, 54-90).

und diese im Gegenzug den Zahlungsempfängern Einlagen gutschreiben. Da Zentralbanken als Schöpferinnen der Währung agieren, können sie ihre Ausgaben gar nicht „finanzieren" – es entsteht immer neues Geld (sowohl Einlagen wie auch Zentralbankguthaben), wenn sie Ausgaben im Auftrag der Regierung tätigen. Dies gilt auch für Deutschland innerhalb der Eurozone: Die Bundesbank tätigt alle Ausgaben der Bundesregierung in deren Auftrag.[2]

Lediglich auf der Ebene der politischen Regeln lässt sich eine „Finanzierung" von Staatsausgaben konstruieren. So muss das Zentralkonto des Bundes (bei der Bundesbank), das bei Staatsausgaben belastet wird, am Ende des Tages ausgeglichen sein. Dieses Konto wird durch Steuereinnahmen und Verkäufe von Staatsanleihen gefüllt. Es handelt sich dabei allerdings nicht um „Geld" im physischen Sinne, sondern lediglich um einen Punktestand zur Verrechnung von Einnahmen und Ausgaben. Nur wenn der Punktestand nicht negativ ist, kann die Bundesbank die Ausgaben der Bundesregierung in deren Auftrag tätigen, indem sie das Zentralbankkonto einer Bank erhöht und das der Bundesregierung reduziert. Aus rein technischer Sicht ist dies so aber nicht notwendig – wer in einer Bilanz einen Eintrag erhöhen will, muss die Zahlen, die dort eingetragen werden, offensichtlich nicht im Vorfeld „ansparen".

Der Staat (inklusive seiner Zentralbank) schöpft seine Währung nach eigenen politischen Regeln. Da er nichts weiter verspricht, als die Währung zur Begleichung von Steuerschulden sowie für alle weiteren Zahlungen an den Staat anzunehmen, handelt es sich letztlich um Steuergutschriften (Ehnts und Paetz, 2019). Der Staat gibt auf Bundesebene Geld aus und entzieht es im Nachhinein über Steuerzahlungen dem Kreislauf. Da ein Staat mit eigener Währung seine Zahlungen immer leisten kann, sollten Defizite daher auch nicht die Grundlage wirtschaftspolitischer Regeln darstellen (Kelton, 2020). Staatliche Ausgabenüberschüsse gehen zwangsläufig mit gleichhohen Einnahmeüberschüssen im privaten Sektor einher.[3] Statt von einem staatlichen Defizit könnte man daher auch von einem Überschuss im Privatsektor sprechen. Eine alternative Definition von Staatsverschuldung wäre dann: „Im Besitz des Privatsektors befindliche Steuergutschriften."

Die Leistungsfähigkeit einer Volkswirtschaft wird lediglich von den vorhandenen Ressourcen eingeschränkt – nicht durch vorhandene Zahlungsmittel. Dies bedeutet natürlich nicht, dass alle nutzbaren Ressourcen auch verbraucht werden sollten. Wofür und in welchem Umfang die vorhandenen Möglichkeiten genutzt werden sollten, hängt von den wirtschaftspolitischen Zielen ab. Diese sind nach Mosler (1997) Vollbeschäftigung und Preisstabilität und sollten um eine nachhaltige Ressourcennutzung ergänzt werden.

Die Inflationsrate wird im Wesentlichen durch die Entwicklung der Lohnstückkosten bestimmt. Nähert sich die Volkswirtschaft der Vollbeschäftigung ist zu erwarten, dass die Löhne schneller steigen werden und die Lohnstückkosten in die Höhe treiben. Bei Unterauslastung ist hingegen nicht mit einer Beschleunigung der Preisentwicklung zu rechnen. Die Inflationsrate kann grundsätzlich durch eine höhere Arbeitslosigkeit verringert werden, hängt letztlich aber von den Lohnverhandlungen zwischen Angestellten und Arbeitgeber:innen ab. Die Höhe der Beschäftigung wird wiederum von der effektiven Nachfrage bestimmt.

Private Ausgaben hängen im Wesentlichen von langfristigen Erwartungen ab. Da der Einfluss der Zinsen auf die Investitionsgüternachfrage eher untergeordnet ist, ist die Zentralbank mit der Stabilisierung einer Volkswirtschaft überfordert und benötigt die Unterstützung der Fiskalpolitik. Dies gilt insbesondere in Krisen, in denen naturgemäß die langfristigen Erwartungen getrübt sind.

Der Staat kann die Entwicklung des allgemeinen Preisniveaus auch über die staatlichen Preise unter anderem im öffentlichen Personennahverkehr, im Gesundheitssystem und in anderen Bereichen sowie über den Mindestlohn und die Löhne im öffentlichen Dienst beeinflussen. Weiterhin beeinflusst der gesetzliche Rahmen für die Tarifbindung über die Gewerkschaften die Entwicklung von Lohnstückkosten und Preisen.

Geld-, Fiskal- und Lohnpolitik sollten gemeinsam darauf ausgerichtet sein, die Ziele Vollbeschäftigung und Preisstabilität zu erreichen. Das Ziel stabiler Preise allein der Zentralbank zu übertragen und mit regelmäßiger Austeritätspolitik einen Anstieg der Arbeitslosigkeit zu verursachen, der zu tendenziell sinkenden Inflationsraten führt, ist dagegen nicht zielführend. Da sich die Finanzierungsfrage für eine Regierung mit eigener Währung nicht stellt, ist eine Sparpolitik nicht notwendig, solange die Wirtschaft unterausgelastet ist.

## Aktuelle Entwicklungen bestätigen die MMT-Sicht

Dass eine Regierung in ihrer eigenen Währung nicht zahlungsunfähig werden kann, sofern die Zentralbank kooperiert, ist derzeit besonders deutlich in Großbritanni-

2 Mit der Unabhängigkeit der Zentralbank hat dies nichts zu tun. Die Bundesbank ist Hausbank der Bundesrepublik Deutschland und führt immer alle Zahlungen der Bundesregierung durch.
3 In einer offenen Volkswirtschaft kann ein staatliches Defizit im Inland auch zu Überschüssen im Ausland führen.

en zu erkennen. Die Bank of England (2020) gewährt der britischen Regierung in der Pandemie die Möglichkeit, das sogenannte Ways-and-Means-Konto zu überziehen. Zusätzliche Staatsausgaben sind dann nicht an den Verkauf verzinster Staatsanleihen an den Privatsektor gebunden. Dies verdeutlicht, dass ein Staat dank des Währungsmonopols keiner Einnahmen bedarf, um Ausgaben zu tätigen. Die Emission von Staatsanleihen ist lediglich optional und kann dazu verwendet werden, die Zentralbankguthaben der Banken zu reduzieren, sofern dies erwünscht ist.

In Kanada gibt es diese „direkte Staatsfinanzierung“ bereits seit Jahrzehnten. Die oben skizzierte Befürchtung einer steigenden Inflationsrate trat nicht ein. Becklumb und Frigon (2015, 1-2) zufolge behält die kanadische Zentralbank typischerweise etwa 20 % aller Staatsanleihen ein. Dafür schreibt sie der Regierung auf ihrem Konto den entsprechenden Gegenwert gut. Die Autoren stufen diese Transaktion als „intern“ ein, da Anleihen, die der Staat bei sich selbst hält, genauso wenig eine Verbindlichkeit darstellen wie ein Schuldschein, den man gegen sich selbst ausstellt. Ähnlich argumentiert Andolfatto (2020) von der St. Louis Federal Reserve Bank für die USA. In den Zentralbanken mehren sich seit langem die Stimmen, welche die herkömmliche Sichtweise auf die Staatsfinanzierung infrage stellen.

Auch die Entwicklung in der Eurozone bestätigt die theoretischen Überlegungen der MMT. Ökonomen wie Randall Wray oder Wynne Godley haben von Beginn an kritisiert, dass die strengen Defizitregeln des Euro-Raums einer angemessenen Wirtschaftspolitik im Wege stehen (z. B. Godley, 1992). Tatsächlich ist die Arbeitslosenquote seit Bestehen der Eurozone nie unter 7 % gefallen. Auch das Inflationsziel wurde in den letzten zehn Jahren bestenfalls sporadisch erreicht. Vollbeschäftigung und Preisstabilität hängen sehr viel stärker von den Regierungsausgaben ab, als es die meisten Ökonom:innen wahrhaben wollen.

Dies hat inzwischen wohl auch die europäische Kommission eingesehen und die auf staatlichen Defiziten basierenden Regeln der Eurozone nach Ausbruch der Pandemie zügig außer Kraft gesetzt. Schon im März 2020 wurde die allgemeine Ausstiegsklausel aktiviert, sodass Defizite nicht mehr sanktioniert werden. Ebenfalls im März 2020 nahm die Europäische Zentralbank (EZB) die Rolle des „Kreditgebers der letzten Instanz“ an, indem sie das Pandemic Emergency Purchase Programme (PEPP) auflegte. Dieses gestattet ihr den nahezu unbegrenzten Ankauf von Staatsanleihen, womit deren Ausfallrisiko quasi auf null reduziert wurde. So konnte eine erneute Eurokrise mit steigender Verzinsung der Staatsanleihen aus der Peripherie vermieden werden.

## Schulden, Zinsen und Wachstum

Im Handelsblatt fordert Tom Krebs (2020) eine europäische Fiskalregel, die den Verschuldungsgrad einschränkt. Der englische Economist (2020) hingegen fordert in einem Leitartikel, die Defizitgrenzen von der Arbeitslosenquote abhängig zu machen und sie im Falle erhöhter Arbeitslosigkeit auszusetzen. Auch Blanchard et al. (2020) plädieren für eine Modifikation der europäischen Fiskalregeln, weil die Dynamik von Schuldenquoten zu komplex sei, um sie anhand einer einzigen Kennzahl (der Schuldenquote) zu beurteilen. Sie fordern qualitative Standards, um einen explosiven Pfad der staatlichen Schuldenquote zu verhindern, sowie stärker länderspezifische Analysen und einen größeren fiskalpolitischen Spielraum.

Auch diese Analyse – obgleich sie einen deutlichen Schritt in die richtige Richtung macht – greift zu kurz. Ein explosiver Schuldenpfad ist aus unserer Sicht ausgeschlossen, sofern die Zentralbank kooperiert. Üblicherweise wird argumentiert, dass für die langfristige Stabilität der Schuldenquote die Wachstumsrate des BIP über dem Zins für Anleihen liegen muss. Abbildung 1 zeigt, dass dies bis in die 1970er Jahre die Regel war. Nachdem die Ölpreiskrisen die Inflationsraten in den meisten Industrienationen deutlich ansteigen ließen, entschieden sich die Zentralbanken jedoch dazu, bereits jedes noch so kleine Anzeichen von Inflation mit stark ansteigenden Zinsen zu bekämpfen. In Kombination mit dem Rückgang staatlicher Ausgaben zur Stabilisierung der Beschäftigung führte dies zu Jahrzehnten, die von steigender Arbeitslosigkeit und fallenden Wachstumsraten geprägt waren.

Nach den Finanzkrisen von 2001 und 2007/2008 sanken die Zinsen in den meisten Nationen dann aber wieder unter die Wachstumsrate. Die Zentralbanken drückten mit Anleihekäufen die langfristigen Zinsen, da man sich nur so in der Lage sah, die große Rezession zu bekämpfen. Da die Fiskalpolitik im Euroraum in den darauffolgenden Jahren aber wieder dem Austeritätskurs folgte und fallende Zinsen in einem von Rezession geprägten Umfeld keinen nennenswerten Anstieg der Investitionsgüternachfrage hervorbringen, hat sich die Eurozone selbst bei negativen Zinsen nicht von der Finanzkrise erholen können (Ehnts und Paetz, 2021).

Die Befürwortenden geringer Schuldenquoten befürchten, dass die Zinsen auf staatliche Schuldverschreibungen steigen werden, sobald sich die Verschuldung im Verhältnis zum BIP erhöht. Abbildung 2 zeigt, dass es in der Vergangenheit einen solchen Anstieg trotz steigender Schuldenquoten aber nicht gegeben hat. Die Gründe hierfür sind zum einen die geringeren Inflationsraten, die zu einem fallenden Zinsniveau geführt haben, sowie das (im-

Abbildung 1

**Differenz zwischen Zinsen für 10-jährige Staatsanleihen und BIP-Wachstumsrate für ausgewählte Länder**

Gleitende Dreijahresdurchschnitte in Prozentpunkten

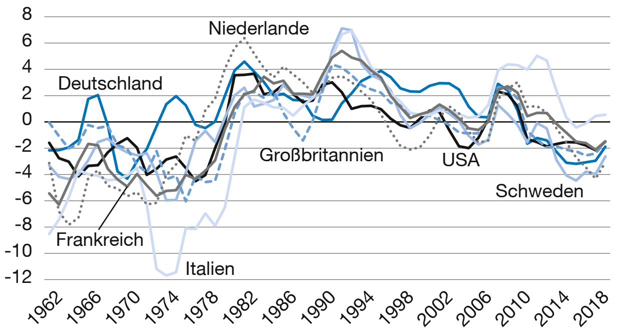

Quelle: Ameco.

plizit gegebene) Signal der Zentralbanken, staatliche Anleihen notfalls unbegrenzt aufzukaufen. Letzteres hat den Käufer:innen staatlicher Anleihen versichert, dass ihr Geld sicher angelegt ist und so die Risikoaufschläge aufgrund eines möglichen Zahlungsausfalls eliminiert. Dennoch bestünde prinzipiell die Gefahr, dass bei wieder steigenden Zinsen die Schuldenquoten einem explosiven Pfad folgen würden. Die Zinsen werden aber nur wieder ansteigen, nachdem die Krise überwunden wurde und daher auch die Wachstums- wie Inflationsraten wieder ansteigen.

## Fiskalische Nachhaltigkeit neu denken

Kommentatoren wie This is money (2020) argumentieren zudem, dass Anleihen im Besitz der staatlichen Zentralbank nicht mehr zu den Staatsschulden gerechnet werden sollten. Schließlich zahlt die Regierung die Zinsen sowie die Tilgungsraten an die eigene Zentralbank.[4] Berücksichtigt man diesen Punkt, muss auch die Stabilitätsbedingung überdacht werden. Beim Ankauf staatlicher Anleihen erhöht die Zentralbank die Reserveguthaben der Geschäftsbanken. Da die (langfristigen) Zinsen auf Staatsanleihen in der Regel über den Zinsen liegen, welche die Zentralbank für die Reserveguthaben der Banken zahlt, erhöht sich hierdurch ihr Gewinn, der wiederum an die Regierung zurückgezahlt wird. Eine stabile Schuldenquote kann daher auch erreicht werden, wenn die Zinsen auf Staatsanleihen über der Wachstumsrate liegen, weil ein Teil der Zinszahlungen ohnehin wieder an die Regierung zurückfließt. Des Weiteren entspricht die Schuldenquote, die lediglich die Anleihen in Besitz des Privatsektors berücksichtigt, nur dem Anteil der nicht von der Zentralbank gehaltenen Anleihen an der gesamten Schuldenquote.[5]

Unabhängig von der Höhe der Schuldenquote hängt die Zahlungsfähigkeit einer Regierung ausschließlich von den politischen Regeln ab, die auf europäischer Ebene auf Basis des Konzepts der „fiskalischen Nachhaltigkeit“ verfasst werden (Fullwiler, 2016, 14). Hierzu werden Referenzwerte für Defizite (3 % des BIP) und Schuldenstand (60 % des BIP) vorgegeben. Ein robuster negativer Zusammenhang zwischen der Höhe der Staatsverschuldung und der realen Wachstumsrate einer Volkswirtschaft konnte bisher aber nicht nachgewiesen werden (Breuer und Colombier, 2020). Da eine Zahlungsunfähigkeit zudem ausgeschlossen ist, solange die Zentralbank ihre Funktion als Kreditgeber der letzten Instanz erfüllt, sind die europäischen Vorgaben für Defizite und Schuldenquoten sowohl willkürlich als auch unnötig. Da die wirtschaftliche Entwicklung auch von den staatlichen Ausgaben beeinflusst wird, begrenzen die Referenzwerte de facto das Wirtschaftswachstum.

Aktuell sorgt die EZB mit Käufen auf dem Sekundärmarkt dafür, dass Staatsanleihen als risikofrei angesehen werden. In Kombination mit dem Aussetzen des Stabilitäts- und Wachstumspakts (SWP) gibt es daher derzeit de facto keine Ausgabenbegrenzungen für die Regierungen der Eurozone. Allerdings ist eine Rückführung der Verschuldung auf die EU-Richtwerte bereits angedacht. Der Stabilitätsrat (2020, 2) sagt voraus: „In den Jahren 2022 und 2023 wird mit dem erwarteten Abbau des gesamtstaatlichen strukturellen Finanzierungsdefizits um jeweils mehr als ½ % des BIP der Richtwert der europäischen Haushaltsüberwachung für eine Rückführung des strukturellen Defizits erfüllt.“ Da staatliche Überschüsse bei gegebener Handelsbilanz mit privaten Defiziten einhergehen müssen, kann diese Strategie nicht aufgehen, sollte der Privatsektor nach der Pandemie aufgrund einer höheren Verschuldung (bzw. geringerer Ersparnisse) ebenfalls seine Ausgaben reduzieren wollen. In diesem Fall droht ein weiteres verlorenes Euro-Jahrzehnt, sofern staatlicher wie privater Sektor gleichzeitig versuchen, ihre Ausgaben zu senken.

## Die Zukunft der Eurozone

Um den Regierungen der Eurozone die nötigen Ausgaben zu erlauben, damit ein weiteres selbstverschuldetes Dahinsiechen der Eurozone verhindert werden kann, muss die EZB dauerhaft die Rolle des Kreditgebers der letzten

4 Dies steht im Einklang mit Postkeynesianischen stock-flow-konsistenten Modellen, in denen die finanziellen Beziehungen einer Volkswirtschaft mit Hilfe der doppelten Buchführung konsequent abgebildet werden (Godley und Lavoie, 2007).

5 Um die Zinsen niedrig zu halten, reicht es zudem in der Regel aus, glaubhaft zu versichern, dass man die Funktion des Kreditgebers der letzten Instanz erfüllt, wie sich z. B. an den sinkenden Zinsdifferenzialen der Staatsanleihen im Euroraum nach der Ankündigung Mario Draghis, alles zu tun, um die Eurozone zu stabilisieren, sowie der Auflage des OMT-Programms der EZB nach 2012 erkennen lässt.

Abbildung 2

**Zinsen für 10-jährige Staatsanleihen und Schuldenquoten für ausgewählte Länder**

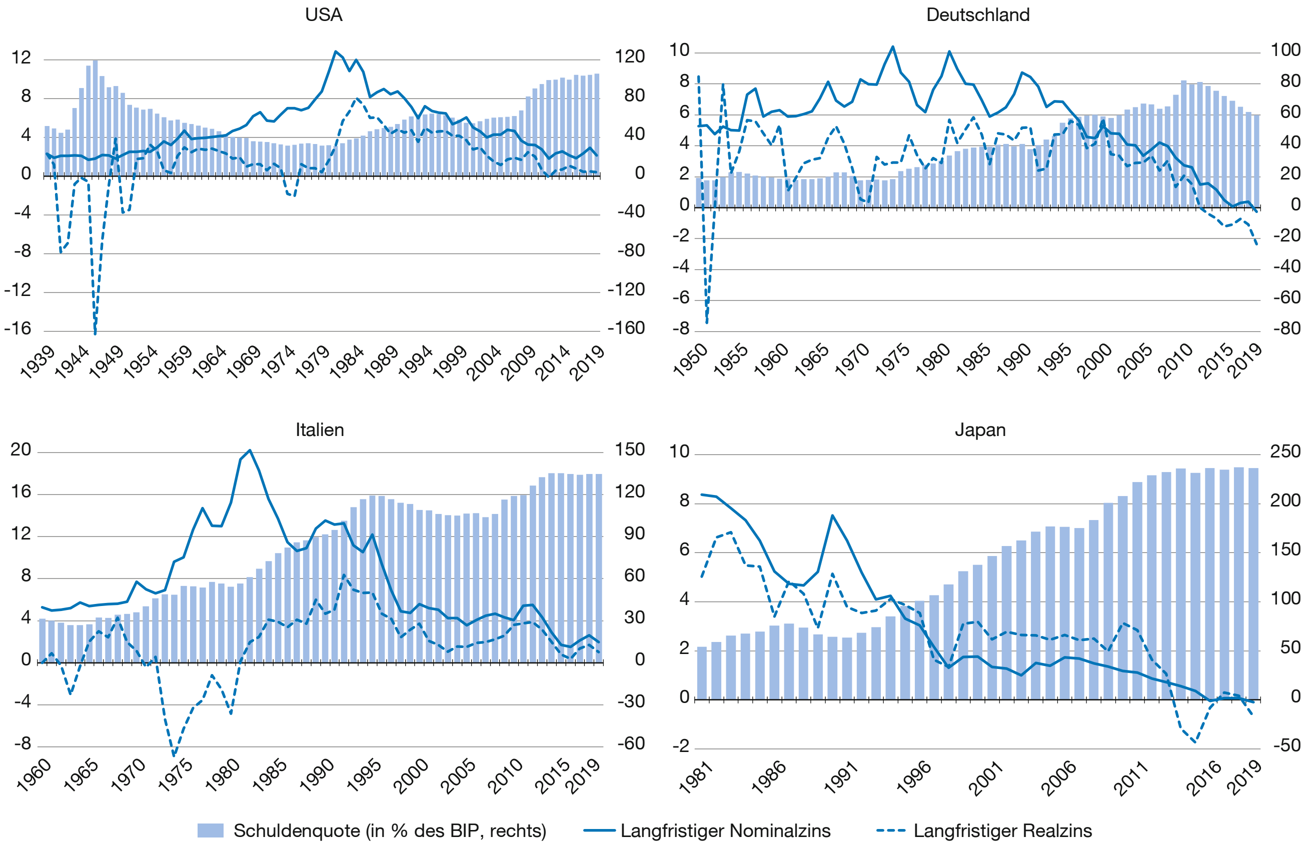

Quellen: Ameco und FRED; eigene Berechnungen.

Instanz wahrnehmen. Hierzu könnte z. B. das PEPP-Programm verstetigt werden. Eine Rückkehr der Zinsdifferenzen und der entsprechenden Dynamik mit dem Ende eines partiellen Zahlungsausfalls wie bei Griechenland würde hingegen eine Abwärtsspirale erzeugen, die einige Mitgliedstaaten zu einem Austritt aus dem Euroraum bewegen könnte.[6] Wenn die Rolle des Kreditgebers der letzten Instanz zu den offiziellen und permanenten Aufgaben der EZB gehört, wäre eine staatliche Insolvenz der Mitgliedstaaten der Eurozone – und somit auch die Haftungsfrage – hingegen ein für alle Mal ausgeschlossen. Damit wären auch die Zinsaufschläge sowie der „Bank-Sovereign Doom Loop" für immer verschwunden.

Sollte der SWP jedoch eingesetzt werden, bevor BIP und Arbeitslosenquoten in allen Mitgliedstaaten wieder das Vorkrisenniveau erreicht haben, würde dies zeigen, dass EU-Kommission und -Rat weiterhin den alten Denkmustern folgen. Dies gilt ebenso für die vielen nationalen Schuldenbremsen, die einer expansiven Fiskalpolitik im Wege stehen und abgeschafft werden sollten. Des Weiteren sollte von der Auflage „struktureller Reformen" abgesehen werden, so wie sie im Rahmen des Next Generation Programms unter anderem von der spanischen Regierung gefordert werden (El Pais, 2020). Die EU-Kommission hat mit ähnlichen Auflagen bereits in den vergangenen Jahren viel Vertrauen verspielt. Im Rahmen ihrer Austeritätspolitik in den 2010er Jahren wurde der italienischen Regierung z. B. dutzendfach empfohlen, die Ausgaben im Gesundheitsbereich zu reduzieren.

Diese Krise sollte daher nicht dazu genutzt werden, weitere Kompetenzen nach Brüssel zu verlagern, um so eine erneute Sparpolitik zu erzwingen und den Vertrauensverlust in die europäischen Institutionen zu verstärken. Im Rahmen der Bekämpfung der Corona-Pandemie wäre es stattdessen sinnvoll, nationale Ausgaben dafür einzusetzen, Impfstoffe entwickeln zu lassen und über das Gesundheitssystem an die Bevölkerung abzugeben. Da die EZB kostenlos und unbegrenzt Euros erzeugen kann, liegen die Grenzen dabei in den realen Ressourcen, nicht jedoch im vermeintlich knap-

6 Zudem sind Zinsdifferenzen schwerliche mit einem fairen Wettbewerb der Euro-Mitgliedstaaten vereinbar (Kaczmarczyk, 2021).

pen Geld. Der Indikator, an dem man sich stattdessen in Zukunft orientieren sollte, ist die Arbeitslosenquote.

### Neue Geld- und Fiskalpolitik

Seit den Ölpreiskrisen haben die Zentralbanken einen wirtschaftlichen Aufschwung bei jeglichem Verdacht auf steigende Inflationsraten über eine Erhöhung der Zinsen sofort abgewürgt. Während der „Zinshammer“ effektiv war und die Inflationsraten fielen, kann dies von den Zinssenkungen jedoch nicht behauptet werden. Sie feuerten die Vermögenspreise an, führten aber nicht zu einer ausreichenden Erholung am Arbeitsmarkt. So stiegen die Arbeitslosenquoten mit jedem Konjunkturzyklus weiter.

Dagegen wäre eine „sanfte“ Steuerung sinnvoller, solange kein stetiger Anstieg der Inflationsrate zu erkennen ist. Statt sofort auf jeden Aufschwung mit drastischen Zinserhöhungen der Zentralbank zu reagieren, kann der Staat durch die Zurückstellung von Investitionen die Nachfrage reduzieren oder Lohnerhöhungen für staatliche Angestellte verringern. Die automatischen Stabilisatoren dämpfen die Inflationsentwicklung zudem, da im Aufschwung durch höhere Steuereinnahmen mehr Kaufkraft abgeschöpft wird.[7] In der Eurozone wäre es derzeit notwendig, die Stabilisierung der wirtschaftlichen Entwicklung stärker der nationalen Fiskalpolitik zu übertragen, wenngleich auch eine Koordination der Wirtschaftspolitik wünschenswert wäre. Das Inflationsziel von 2 % können wir nur erreichen, wenn die Lohnstückkosten schneller wachsen. Auch hierzu können höhere Staatsausgaben maßgeblich beitragen, wenn sie die Verhandlungsmacht der Arbeitnehmer:innen über Engpässe am Arbeitsmarkt stärken.

### Auswirkungen auf Außenhandel und Wechselkurse

Eine höhere Nachfrage könnte über zusätzliche Importe dazu führen, dass die Nettoexporte der Eurozone fallen. Sollte ein Handelsbilanzdefizit entstehen, wäre dies bei einem flexiblen Wechselkurs jedoch kein Grund zur Sorge. Ein Anstieg von Verbindlichkeiten in Fremdwährung könnte schlimmstenfalls zu einer Abwertung des Euros führen, die dem Defizit tendenziell entgegenwirken würde. Da diese Fremdwährungsverbindlichkeiten im Privatsektor entstehen, ist die Schuldentragfähigkeit der Regierungen hiervon nicht betroffen. Da der Euro gegenüber den Währungen der Handelspartner bisher problemlos schwankte, ohne eine ernsthaft störende importierte Inflation zu induzieren, wird eine expansivere Wirtschaftspolitik keine nennenswerten Auswirkungen auf die Inflation haben.

7 Die MMT vertritt nicht die Idee, dass der Staat im Aufschwung die Steuersätze oder das Steueraufkommen erhöht. Dennoch lassen sich Fehlentwicklungen auch durch gezielte Steuerhöhungen bremsen.

Dullien und Tober (2019) argumentieren hingegen, dass „[w]enn allerdings der Staat seinen Schuldendienst kontinuierlich mit neu geschaffenem Geld deckt, [...] eine kontinuierliche Ausweitung der Geldmenge ... über Wechselkurseffekte portfoliotheoretisch eine kontinuierliche Abwertung bedeuten“ würde. Vier Argumente sprechen gegen diese Sicht. Wie bereits gezeigt, kann *erstens* die Zentralbank einen explosiven Pfad der Schuldenquote jederzeit verhindern. Die Deckung des Schuldendienstes für die Anleihen, die sich im Besitz der Zentralbank befinden, ist lediglich ein interner Buchungsvorgang, von dem die Geldmenge im Privatsektor nicht betroffen ist. *Zweitens* korrelieren die Entwicklungen von Geldmenge und Wechselkursen nicht in der behaupteten Weise. *Drittens* gehören zu einem Wechselkurs immer zwei Länder. Sollten die staatlichen Defizite in anderen Länder gleichermaßen ansteigen (was keineswegs unrealistisch ist), wäre daher ohnehin kein Effekt zu erwarten. *Viertens* hängt die Entwicklung der Geldmenge von einer Vielzahl von Faktoren ab. Sollte der Privatsektor nach der Pandemie die Einnahmen aus einer Erhöhung der Staatsausgaben z. B. nutzen, um Kredite zurückzuzahlen, die während der Krise aufgenommen werden mussten, würde Geld vernichtet und einem Anstieg der Geldmenge entgegengewirkt werden. Die Währungen von Norwegen, Schweden und Dänemark zeigen exemplarisch, dass hohe Staatsausgaben nicht zwangsläufig mit einer Abwertung der Währung einhergehen müssen. Eine „kontinuierliche Abwertung“ wäre demnach nicht zu erwarten, wenn die Eurozone sich zu einem Green New Deal durchringen würde. Sollte eine Regierung ihre Ausgaben jedoch auch bei Vollauslastung permanent weiter erhöhen, ist zwangsläufig mit einer Inflation zu rechnen, die zu einer Abwertung führen kann. Dies ist aber keine Politik, die von MMT-Befürwortenden vertreten wird.

### Sozio-ökologische Transformation demokratisch gestalten

Mit Hinblick auf den Klimawandel und dessen Folgen erscheint es nicht geboten, den Einsatz von Rohstoffen und Energie weiter zu intensivieren. Der Staat sollte daher lenkend und koordinierend tätig werden und darf sich dabei nicht vom Blick auf Defizite und Schuldenhöhe leiten lassen. Ohne höhere Staatsausgaben werden wir die notwendige ökologische Transformation nicht bewältigen können. Es gilt, die Ideologien des 20. Jahrhunderts zu überwinden. Weder Staat noch Markt allein werden unsere Probleme lösen können. Zudem gilt es die Gesellschaft wieder zu einen, die derzeit an der zunehmenden Ungleichheit zu zerbrechen droht. Die mit der wirtschaftlichen Schieflage einhergehende soziale Ausgrenzung führt zu mehr Aggression und Gewalt (Bauer, 2011).

Das Overton Window scheint, wie schon 2009/2010, wieder offen zu stehen. Eine Wiedereinsetzung der Maastricht-Regeln in Verbindung mit einer Schuldenrückführung auf Basis nationaler Schuldenbremsen würde Deutschland wie auch die Eurozone innerhalb weniger Jahre in eine Rezession oder gar Depression stürzen. Letzteres ist besonders dann zu befürchten, wenn das PEPP der EZB beendet wird und die Zinsen der Staatsanleihen südeuropäischer Länder wieder steigen würden. Vor diesem Hintergrund gilt es, eine adäquate makroökonomische Steuerung zu etablieren statt sich mit der sinnlosen Frage der Finanzierung staatlicher Ausgaben zu blockieren. Die MMT kann als Beschreibung unseres Geldsystems dabei helfen, einen solchen Weg zu finden. Genauso wie Umweltschützer und Ingenieurinnen die Funktionsweise eines Benzinmotors verstehen sollten, sollten auch Politikerinnen und Bürger das Geldsystem verstehen. Nur so kann auf demokratischem Wege aus allen Möglichkeiten zur Gestaltung der Zukunft der gewünschte Pfad ausgewählt werden.
